# Genetic Mapping of a QTL Controlling Fruit Size in Melon (*Cucumis melo* L.)

**DOI:** 10.3390/plants14152254

**Published:** 2025-07-22

**Authors:** Fazle Amin, Nasar Ali Khan, Sikandar Amanullah, Shusen Liu, Zhao Liu, Zhengfeng Song, Shi Liu, Xuezheng Wang, Xufeng Fang, Feishi Luan

**Affiliations:** 1 Key Laboratory of Biology and Genetic Improvement of Horticulture Crops (Northeast Region), Ministry of Agriculture and Rural Affairs, College of Horticulture and Landscape Architecture, Northeast Agricultural University, Harbin 150030, China; fazleamin204@gmail.com (F.A.); nasaralikhan001@gmail.com (N.A.K.); shiliu@neau.edu.cn (S.L.); xz6206815@163.com (X.W.); 2 College of Horticulture and Landscape Architecture, Northeast Agricultural University, Harbin 150030, China; 3 Department of Horticultural Science, North Carolina State University, Mountain Horticultural Crops Research and Extension Center, 455 Research Drive, Mills River, NC 28759, USA; sikandaraman@yahoo.com; 4 Watermelon and Melon Breeding Engineering Technology Research Center of Shandong Province Co., Ltd., Weifang 261000, China; sanmuseed@163.com (S.L.); sanmuliuzhao@163.com (Z.L.); wdlszf@163.com (Z.S.)

**Keywords:** melon, fruit length, fruit diameter, molecular markers

## Abstract

Fruit size is an important agronomic trait affecting the yield and commercial value of melon and a key trait selected for during domestication. In this study, two respective melon accessions (large-fruited M202008 and small-fruited M202009) were crossed, and developed biparental mapping populations of the F_2_ generation (160 and 382 plants) were checked across two subsequent experimental years (2023 and 2024). The phenotypic characterization and genetic inheritance analysis showed that melon fruit size is modulated by quantitative genetics. Bulked segregant sequencing analysis (BSA-seq) identified a stable and effective quantitative trait locus (QTL, named *Cmfs*) controlling fruit size, localized to a 3.75 Mb region on chromosome 9. To better delineate the main-effect *Cmfs* locus, co-dominant polymorphic molecular markers were developed in this genetic interval, and genotyping was performed within the F_2_ mapping populations grown across two years. QTL analysis of the phenotypic and genotypic datasets delimited the major-effect *Cmfs* locus interval for fruit length [2023: logarithm of odds (LOD) value = 6.16, 16.20% phenotypic variation explained (PVE); 2024: LOD = 5.44, 6.35% PVE] and fruit diameter (2023: LOD value = 5.48, 14.59% PVE; 2024: LOD = 6.22, 7.22% PVE) to 1.88 and 2.20 Mb intervals, respectively. The annotation analysis across the melon genome and comparison of resequencing data from the two parental lines led to the preliminary identification of *MELO3C021600.1* (annotated as cytochrome P450 724B1) as a candidate gene related to melon fruit size. These results provide a better understanding for further fine mapping and functional gene analysis related to melon fruit size.

## 1. Introduction

Melon (*Cucumis melo* L., 2n = 24), an economically important member of the Cucurbitaceae family, is grown worldwide in tropical to temperate zones [1]. The genus originated in Africa, and its center of diversity ranges from Turkey to Japan [2]. China is the leading melon-producing country, followed by India, Turkey, Kazakhstan, and Brazil [3]. The commercial value of melon crops depends strongly on the fruit quality; alongside the shape, texture, and color, the fruit size crucially influences the crop’s utility and yield and consumer preferences. Thus, it is a focal trait in melon breeding [4].

Melons range in size from very small (<100 g) to small (100–400 g), medium (400 g^−1^ kg), large (1–5 kg), and gigantic (4–10 kg). Their shapes range from round to slightly flat, obovoid, ellipsoid, long, and extremely long [5]. It has been known that fruit size is genetically programmed during early floral meristem development. Perin et al. [6] identified a total of 19 fruit morphology-related and 15 ovary-size-related QTLs in two melon populations. The co-segregation patterns of these loci suggested early developmental control of fruit shape [7]. It was stated that the fruit size regulation involves complex interactions among cellular processes including cell cycle dynamics, cell wall metabolism, and plant hormone signaling [8]. To date, more than 200 QTLs related to melon fruit development have been reported [9]. Among these, 78 consensus QTLs and the QTL intervals were found to contain large numbers of homologous genes regulating fruit size [5]. Most of them, including *fsqs12.1* [6], *mfl12.1*/*mfs12.1* [10], *fdqs12.1*/*fsqs12.2* [9], and *FSQM12* [11], are located on chromosome 12. *CmOFP13* (OVATE-domain family protein), located on chromosome 8, is the only gene regulating melon fruit size that has been cloned [12].

Fruit-size-related QTLs in other Cucurbitaceae crops have been cloned successfully. Dou et al. [13] conducted fine mapping of the watermelon fruit-size-related locus *ClFSI3.3* and suggested that *ClSUN25-26-27a* was the key candidate gene. *CsSUN*, the homologous gene in cucumber, induces fruit elongation by promoting longitudinal and inhibiting transverse cell division [14]. *CsTRM5* also regulates cucumber fruit length by interfering with the cell division direction and cell expansion [15]. *CsCRC*, *CsAPR1*, *CsFUL1*, *CsSF1*, and *CsSF2* are other key genes regulating cucumber length; *CsCRC* and *CsAPR1* positively regulate cell enlargement [16], *CsFUL1* influences the auxin content [17], and *CsSF1* and *CsSF2* regulate cytokinin signaling and gibberellin synthesis to reduce the number of fruit cells and the formation of small cucumbers [18]. In addition, cytochrome P450s are one of the largest enzymatic protein families found in many plants. They are involved in the growth and development of organs and have been found to play key roles in the regulation of watermelon, tomato, and sweet cherry fruit size [19,20,21]. They regulate auxin biosynthesis [22], are involved in gibberellin metabolic pathways [23], and participate in the regulation of brassinosteroid biosynthesis [24]. Brassinosteroids also play critical roles in various plant growth processes, including the regulation of cell proliferation and elongation [25].

QTL mapping is a key molecular technique in breeding and genetics studies that helps in evaluating the regulatory basis of crop-specific traits. The construction of genetic linkage maps is a critical prerequisite for the identification of stable QTL regions. The basic genetic maps were first developed in the 1980s and 1990s, and they usually included a minimum of 10–100 markers, e.g., SSR, AFLP, and RAPD [26,27]. However, conventional markers provided required extensive labor, provided limited genomic coverage, and restricted resolution. Later, the release of the melon genome in 2012 [28] laid the foundation for high-resolution genetic mapping of the species. During the last decade, the advanced multi-omics approaches that utilize next-generation sequencing technologies for whole genome sequencing have become more appealing to researchers, which has enabled them to produce ultra-high-density genetic maps of melon using single-nucleotide polymorphism (SNP) markers and more accurate genotyping [29].

High-density genetic mapping at resolutions of up to hundreds of kilobases [30,31] has been performed for many Cucurbitaceae family members, e.g., cucumber [32], squash [33], bitter gourd [34,35], watermelon [36], and melon [29,37,38]. In this study, an F_2_ melon population was obtained by crossing the different accessions of small-fruited (M202008) and large-fruited melons (M202009). A major stable QTL was identified on chromosome 9 based on BSA-seq and genetic linkage analysis of fruit length and diameter in combination with genotypic data from a 2-year period. Our comparison of the resequencing results from the two parental lines led us to hypothesize that *MELO3C021600.1* (annotated as cytochrome P450 724B1) is a candidate gene governing melon fruit size. Our study aims to provide useful germplasm resources for the fruit size breeding of melon and to lay the molecular genetic foundation for the study of fruit size in other cucurbit crops.

## 2. Results

### 2.1. Phenotypic Variations in Length and Diameter of Melon Fruits

**Figure 1:**
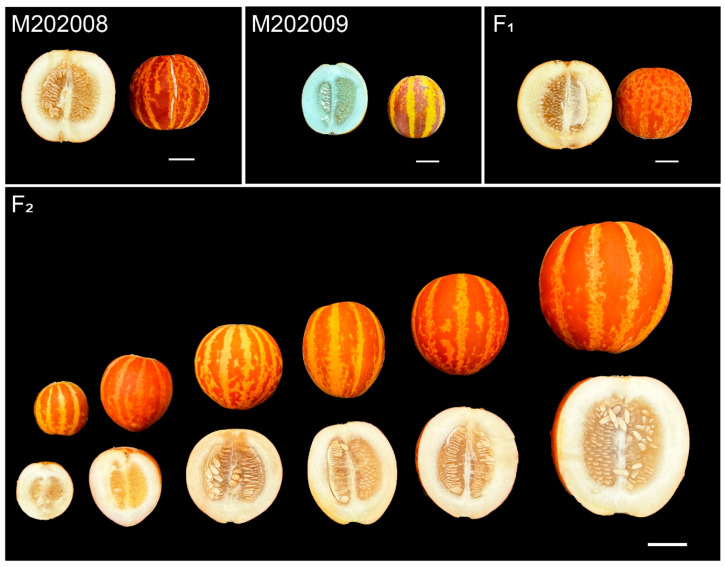
Melon fruit sizes in two distinct parent lines (M202008 and M202009); F_1_ and biparental F_2_ generation. Bar = 3 cm.

**Figure 2:**
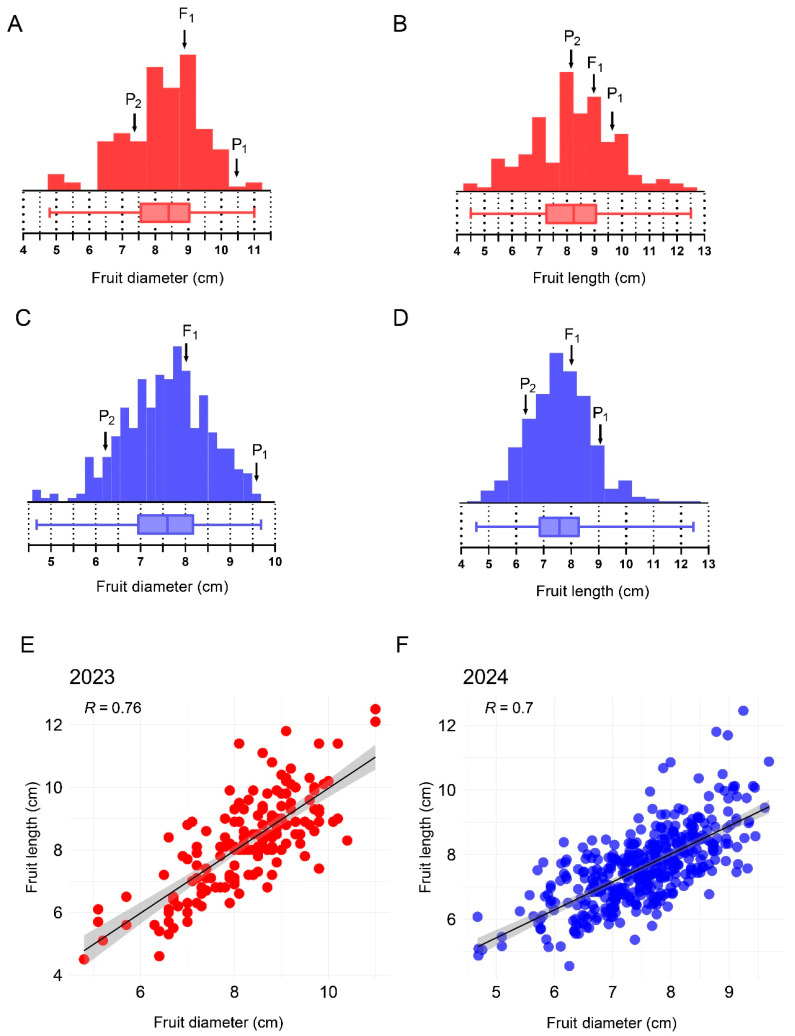
Frequency distributions and correlation analysis of fruit-size-linked traits. (**A**) Frequency distribution for fruit diameter recorded in the F_2_ population grown during 2023. (**B**) Frequency distribution for fruit length recorded in the F population grown during 2023. (**C**) Frequency distribution for fruit diameter recorded in the F population grown during 2024. (**D**) Frequency distribution for fruit length recorded in the F_2_ population grown during 2024. (**E**) Correlation between the fruit length and diameter recorded in the F_2_ population during 2023. (**F**) Correlation between the fruit length and diameter recorded in the F_2_ population during 2024.

The melons produced by the M202009 line were significantly smaller than those produced by the M202008 line (Figure 1). However, the melons of the M202008 line had a mean diameter of 9.80 ± 0.30 cm and length of 9.30 ± 0.60 cm, whereas M202009 melons had a mean diameter of 6.10 ± 0.70 cm and a length of 6.40 ± 0.50 cm. The F_1_ fruits had a mean diameter of 8.10 ± 0.30 cm and a length of 8.10 ± 0.70 cm. For the F_2_ mapping population planted in the experimental year of 2023, fruit diameters ranged from 4.80 to 11.00 cm (mean, 8.40 ± 1.15 cm) and lengths ranged from 4.50 to 12.50 cm (mean, 8.25 ± 1.51 cm; Table 1, Figure 2). The diameters and lengths of melons from the extended F_2_ population planted in the experimental year of 2024 ranged from 4.60 to 9.60 cm (mean, 7.60 ± 0.95 cm) and from 4.50 to 12.40 cm (mean, 7.57 ± 1.17 cm), respectively (Table 1, Figure 2). Thus, the fruit diameter and length distributions were consistent with the characteristics of quantitative traits. Interestingly, these traits were positively correlated in F_2_ mapping populations grown during 2023 (*r* = 0.76) and 2024 (*r* = 0.70), respectively (Figure 2).

### 2.2. BSA-Seq and Genetic Mapping of Cmfs

**Figure 3:**
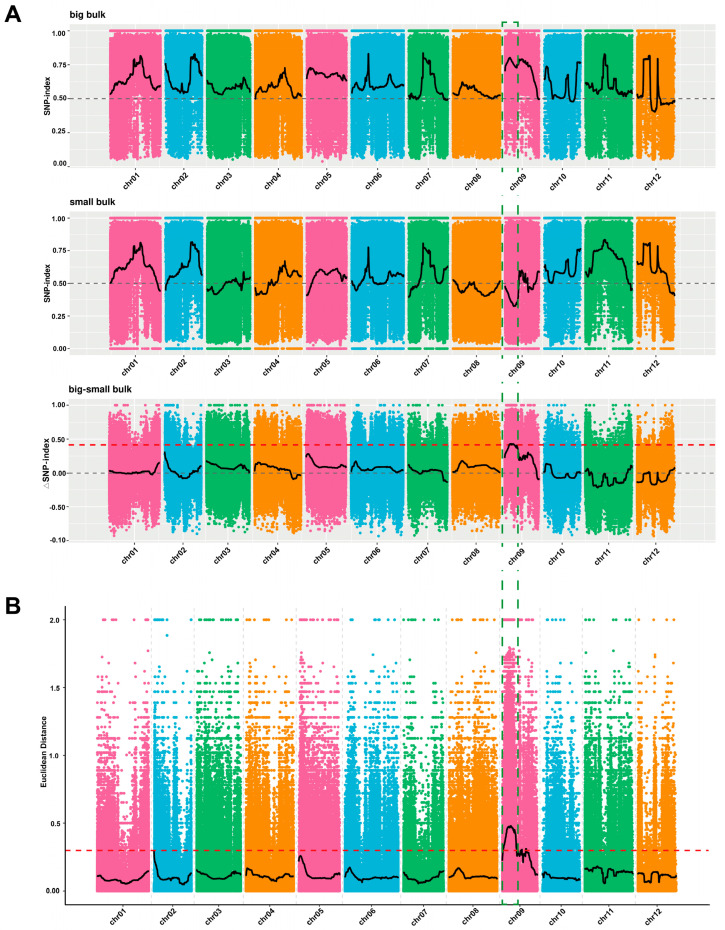
The main-effect genetic regions carrying the melon fruit size signal on chromosome 9, based on two BSA-seq algorithms. (**A**) ∆SNP index for three bulk samples (large, small, and large–small). (**B**) Euclidean distance (ED) values for large–small bulk samples, respectively.

In total, 71,309,728 bp (94.32%) and 78,885,130 bp (96.48%) were obtained for two parental lines (M202008 and M202009), with quality check scores (Q30 values) of 89.31% and 97.38%, respectively. A total of 3,248,677 SNPs were detected after screening the quality and genotype discrepancies between the parental and bulk samples. According to the delta (∆) SNP index and Euclidean distance (ED) values, the main-effect locus regulating melon fruit size (*Cmfs*) was determined to be located within the 3,270,000–7,020,000 bp interval on chromosome 9, spanning about 3.75 Mb (Figure 3).

**Figure 4:**
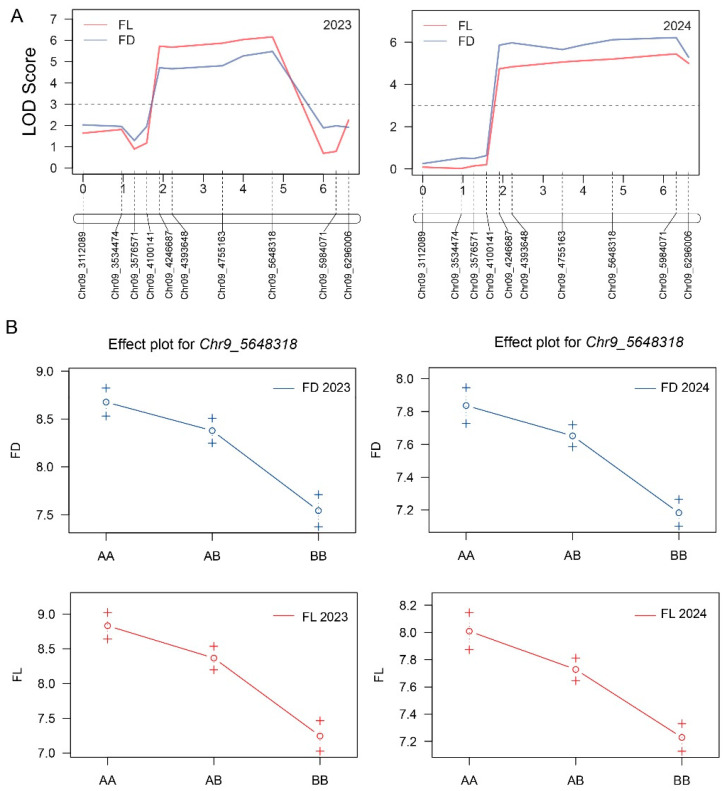
Genetic mapping of melon fruit length and diameter. (**A**) Primary mapping intervals for the F_2_ mapping populations of 2023 and 2024. (**B**) Correlations between phenotypes and genotypes, based on the candidate InDel marker (*Chr9_5648318*). AA, M202008 genotype; AB, heterozygous genotype; BB, M202009 genotype.

Based on the BSA-seq results, a total of 10 polymorphic InDel markers were developed in the target region. The marker genotyping within 2023-F_2_ plants enabled the refinement of the main interval governing the fruit diameter and length to a 1883.93 kb long region between two flanking markers (*Chr9_4100141* and *Chr9_5984071*) (Figure 4A), exhibiting LOD values for the fruit length and diameter of 6.16 and 5.48, with 16.20% and 14.59% of the phenotypic variability, respectively (Table 2). The additional marker genotyping within the 2024-F_2_ population indicated that the targeted region for fruit length and diameter was positioned between adjacent markers (*Chr9_4100141* and *Chr9_6296006*) (Figure 4A), exhibiting LOD values for the fruit length and diameter of 5.44 and 6.22, with 6.35% and 7.22% phenotypic variability, respectively (Table 2). The candidate InDel marker (*Chr9_5648318*) showed the highest LOD value, relative to those with the genotype of the M202008 parent line, and in individuals from the mapping populations of the two experimental years (2023 and 2024).

Further, the genetic effect plot of candidate InDel markers determined the significant and positive correlation between phenotypes and genotypes (AA, M202008 genotype; AB, heterozygous genotype; BB, M202009 genotype). The genotype of the M202009 parent line had shorter fruits (7.20 ± 0.26 vs. 8.80 ± 0.13 cm and 7.20 ± 0.15 vs. 8.0 ± 0.19 cm) and smaller diameters (7.50 ± 0.15 vs. 8.70 ± 0.23 cm and 7.20 ± 0.12 vs. 7.90 ± 0.10 cm).

### 2.3. Candidate Genes Predicted to Be Related to the Cmfs Locus

Based on the annotation analysis of the melon reference genome (DHL92, v4.0), a total of 205 genes were predicted in the mapping region (Table S1). The following gene sequence variations between the parental lines in the coding region of *MELO3C021600.1* were identified: non-synonymous SNP^4,267,567^(C to T), SNP^4,267,818^ (C to T), SNP^4,267,862^ (A to C), SNP^4,268,573^ (A to G), SNP^4,268,604^ (T to A), SNP^4,269,150^ (G to A), and SNP^4,270,637^ (A to G). These variations led to the change of arginine with histidine, aspartic acid with asparagine, phenylalanine with leucine, tyrosine with histidine, leucine with *, and alanine with valine and tyrosine. Additionally, a 3 bp (TGT) insertion was detected at position 4,268,557–4,268,558, leading to a mutation in the *MELO3C021600.1* gene (Table S2). The promoter region of the candidate *MELO3C021600.1* gene was found to contain seven SNPs that differed between parental lines (from the start codon ATG, T→C at 210 and 1027 bp, G→C at 1163 bp, C/T →T at 1211 bp, A/C→A, and G→A at 1742 and 1840 bp; see Table S3). In addition, a 23 bp deletion in the promoter region of *MELO3C021600.1*, potentially affecting its regulatory function, was observed in the M202008 parent line.

## 3. Discussion

Fruit size is a crucial characteristic of melon cultivars. It serves as a key indicator of both fruit quality and yield, and it represents an adaptive feature that has evolved over time in horticultural plants, which are becoming increasingly important for human life. To date, various genes and QTLs have been reported to contribute to fruit enlargement in different horticultural plants during domestication and improvement [39]. This trait has been examined in various cucurbits, including melon [10,40,41], watermelon [19], cucumber [42,43], and tomato [44,45]. However, fruit size is known as a complex polygenic trait, exhibiting clusters of QTLs and genes distributed across the melon genome; however, the underlying molecular mechanisms are not fully understood.

The availability of the melon draft genome has accelerated the genetic mapping studies using segregated biparental populations and led to the identification of numerous QTLs related to fruit size, shape, and weight [46,47,48]. In the present study, two melon lines with small and large fruits were crossed, and the fruit size phenotyping of the F_2_ mapping population showed a normal distribution and quantitative genetics of inherited traits. The BSA-seq analysis of two extreme bulks revealed that a candidate genetic interval of this QTL position was 3.75 Mb long (Figure 3). The genotyping of developed InDel markers was used for genetic mapping, and the analysis narrowed down the candidate region of *Cmfs* to between adjacent markers (*Chr9_4100141* and *Chr9_5984071*) on chromosome 9 (Figure 4). Multiple breeding and genetic studies have shown that melon fruit size is regulated by a main-effect region on chromosome 9, and the QTLs detected in this study correspond to those previously identified to be related to fruit size and shape. In previous studies involving the crossing of distinct parental lines, Amanullah et al. [49] identified fruit width QTLs (*FW9.1* and *FW9.2*) and a fruit weight QTL (*FWT9.1*) on chromosome 9. QTLs related to fruit size traits have also been detected in the same region by crossing the Piel de Sapo and PI 161375 lines [10,50]. Similarly, the crossing of a small-fruited wild tomato species with a large-fruited tomato line led to the identification of QTLs on chromosome 9 related to fruit length and width [51]. In this study, the analysis of QTLs in plants grown in two consecutive years led to the identification of a stable genomic region containing *Cmfs*. Among the genes in the mapping region, *MELO3C021600.1* was the predicted candidate, with mutations in the coding sequence and promoter regions.

In the previous studies, the candidate *MELO3C021600.1* gene, a member of the 724B1 family (CYP724B1) that encodes cytochrome P450, has been predicted to be responsible for the regulation of fruit size [2,20,21,52,53]. Cytochrome P450s form the major enzyme protein family found in many plants; they play vital roles in various metabolic pathways by triggering primary and secondary metabolites that promote plant growth, development, and defense [52,54]. The CYP724B1 subfamily of genes belongs to a cluster in the CYP85 group that is related to brassinosteroid metabolism [55,56]. Cytochrome P450 (CYP724B1) shows homology to enzymes involved in brassinosteroid biosynthesis [57]. In rice and tomato, CYP724B catalyzes C-22 hydroxylation during this process [58,59]. Brassinosteroids are essential plant hormones involved in numerous plant growth and development processes, primarily cell division and expansion, seed germination, the enhancement of shoot branching, stress tolerance, and resistance to various pathogens [60,61]. Cytochrome P40 genes have been determined to be involved in controlling the growth and development of *Arabidopsis thaliana* organs [62,63,64]; the genetic regulation for the development of organ size in tomato [20], wheat [65] and soybean [66]; and the regulation of fruit size in watermelon [19], pear [67], and sweet cherry [21]. These associated genes affect mesocarp cell expansion and proliferation during fruit growth and development, and their silencing results in reduced fruit size [19,21].

Based on the results of this study, including the identification of seven non-synonymous mutations (nsSNPs) in the coding region of *MELO3C021600.1*, a 3 bp (TGT) insertion in the M202009 parent line (Table S2), and a 23 bp insertion in the promoter region of the M202009 parent line (Table S3), we hypothesize that *MELO3C021600.1* is a candidate gene for regulating the *Cmfs* locus. However, although the QTLs for fruit length and fruit width detected in this study both showed significant effects (Table 2), the proportion of phenotypic variation explained indicates that other gene loci may also have an impact on the variation in fruit size. This suggests that the fruit size of melon is regulated by multiple genes, such as *fsqs12.1* located on chromosome 12 [6] and *CmOFP13* on chromosome 8 [12]. Future exploration of the relationships between these loci will help to further understand the genetic decoding of melon fruit size. Moreover, fine genetic mapping and gene functional validation and expression analyses are required to verify the role of the gene prediction made in this study. The present study findings provide a genetic basis for advanced marker-assisted breeding and map-based cloning aimed at the enhancement of melon fruit size traits.

## 4. Materials and Methods

### 4.1. Melon Lines and Development of Mapping Population

In the first experimental year of 2022, two different melon lines M202008 (large-fruited line, P_1_) and M202009 (small-fruited line, P_2_) were chosen as experimental materials based on different fruit sizes. These parental lines were grown and crossed to produce their F_1_ generation and mapping population of the biparental F_2_ generation. In the next year (2023), the parental lines (10 plants with 3 replications), the F_1_ generation (10 plants with 3 replications), and the F_2_ generation (160 plants) were grown to examine the genetic inheritance of the fruit size trait and identify the candidate regulatory genomic region through whole-genome BSA-seq and QTL analysis. Then, in the next year (2024), the plants of the same parental lines (10 plants with 3 replications), the F_1_ generation, and an expanded F_2_ mapping population (382 plants) were grown and used to narrow down the identified genomic region of the candidate fruit-size-related genetic locus (*Cmfs*), and the underlying candidate genes were predicted.

All the field experiments were conducted at the Xiangyang Agricultural and Experimental Farm of Northeast Agricultural University, Harbin, China. To grow better fruit crops, a 12 h day length and a 25 °C average temperature in the spring of 2023 and 2024 were used. The flowers were pollinated at the anthesis stage, and mature fruits were collected at the full maturity stage by observing changes in external skin color and the development of an abscission layer at 35 days after pollination (DAP). The freshly harvested fruits were cut into longitudinal sections, photos were taken, and phenotypes were characterized. Fruit length (FL) and diameter (FD) were measured in centimeters (cm) using an electronic vernier caliper [49] with a precision of 0.01 mm. The replicated data of three freshly harvested fruits from each plant was recorded, and averaged values were characterized.

### 4.2. DNA Isolation and BSA-Seq Analysis

Young leaves were collected from each melon plant and quickly frozen at −80 °C. Total genomic DNA was extracted using a modified cetyltrimethylammonium bromide (CTAB) protocol [68]. The isolated DNA was quantified with a spectrophotometer (840-317400, Thermo, Waltham, MA, USA) and samples with an A260/280 ratio between 1.8 and 2.0 were considered acceptable for experiments. The quantified DNA samples were checked using 1% agarose gel and stored at −20 °C. Twenty bulk DNA samples each from small- and large-fruited plants were selected from the primary F_2_ mapping population grown in 2023 and subjected to whole-genome BSA-seq. Whole-genome resequencing of the two parental lines and two gene pools was performed at 30× coverage using Illumina Novaseq 6000 at Lianchuan Biotechnology Company, Hangzhou, China. Then, high-quality reads were filtered against the latest melon reference genome (http://www.cucurbitgenomics.org/organism/18, accessed on 1 December 2023) using the Burrows–Wheeler Aligner (BWA, v0.7.17) software [69], and SNP variants were called with samtools and bcftools [70]. The ΔSNP index was computed based on the Euclidean distance (ED) algorithm to detect the main chromosomal region associated with the melon fruit length and diameter, as described in previous genetic studies [71,72].

### 4.3. Genetic Marker Development and Primary Mapping

To confirm the BSA-seq-based identification of candidate regions (*Cmfs*), insertion/deletion (InDel) markers with base number differences ≥ 5 were designed in the primary mapped region using the filtered whole-genome sequenced reads from the two parental lines. The primers with the best properties of product length and size were exported from Primer Premier software (version 5.0). The markers were labeled with the chromosome name and physical sequence position (Table S4) and oligo-synthesized.

An optimized polymerase chain reaction (PCR) for the markers was performed using a thermal cycler (model 2720; Applied Biosystems Inc., Hercules, CA, USA). The oligo-synthesized primers were checked with the three testing genotypes of P_1_, P_2_, and F_1_, respectively. After the initial screening, primers showing co-dominant polymorphism (with a 58% success rate) were selected for genotyping within the F_2_ mapping population. The polymorphic InDel markers were used for genotyping with the primary F_2_ mapping population of 2023, and the main-effect locus (*Cmfs*) was mapped. QTL analysis was performed to obtain a stable genomic region containing the locus across the F_2_ mapping populations of both experimental years of 2023 and 2024. The PCR products of the markers were subjected to 8% denaturing polyacrylamide gel electrophoresis with silver staining.

For the genetic mapping and QTL analysis, the respective genotypic and phenotypic coded data were grouped and anchored across chromosome 9. JoinMap (version 4.0; https://www.kyazma.nl/index.php/JoinMap, accessed on 26 December 2023) software was used to determine the genetic positions (centimorgans, cM) of the genotypic InDel markers. QTL analysis was performed using R/qtl software (version 1.5) [73], logarithm of odds (LOD) values were obtained, and phenotypic contribution rates were calculated using the LOD threshold value of >3.0. The phenotypic and genotypic data correlated with the corresponding markers to the highest LOD value were analyzed for the QTL interval.

### 4.4. Candidate Gene Prediction

The candidate genes in the mapped region were predicted, and the functional annotations of identified genes were checked using the online database for the reference melon genome (DHL92, http://www.cucurbitgenomics.org/, accessed on 5 March 2024). SNP mutations in the predicted genes were examined by pairwise comparison with the coding and promoter regions of the parental lines using Integrative Genomics Viewer (IGV, version 2.1.2) software (http://software.broadinstitute.org/software/igv/, accessed on 9 March 2024), and gene sequence analysis was performed using DNAMAN (version 10.0; Lynnon Biosoft, San Ramon, CA, USA).

### 4.5. Statistical Analysis

The recorded replicated data were computed in Microsoft^®^ Excel (version 2010) and significant differences were identified using analysis of variance and R/qtl (version 1.5; https://rqtl.org/index.html, accessed on 3 April 2024). GraphPad Prism (version 8.0; GraphPad Inc., San Diego, CA, USA) was used to analyze the frequency distributions, kurtosis, skewness, and correlations in the fruit length and diameter of the biparental mapping population of the F_2_ generation.

## 5. Conclusions

Herein, our whole-genome BSA-seq-based analysis revealed a major-effect locus (*Cmfs*) controlling the fruit size of melons. QTL mapping using InDel markers revealed a stable genetic region on chromosome 9, and gene annotation analysis predicted *MELO3C021600.1* as a candidate gene for controlling the *Cmfs* locus. Our obtained results revealed a reliable genetic locus and putative gene; however, further research is required for functional validation through gene expression analyses. The findings of the present study illustrated a genetic basis for advanced marker-assisted breeding and map-based cloning aimed at the development of improved melon cultivars with proper fruit size traits.

## Figures and Tables

**Table 1:** Genetic segregation of the F_2_ mapping populations derived from the crossing of two different melon accessions (M202008 and M202009), respectively.

Year	Trait	Mean	SD	Range	Kurtosis	Skewness
2023	Fruit diameter (FD, cm)	8.40	1.15	4.80–11.00	0.39	−0.50
	Fruit length (FL, cm)	8.25	1.51	4.50–12.50	0.08	0.04
2024	Fruit diameter (FD, cm)	7.60	0.95	4.60–9.60	−0.04	−0.29
	Fruit length (FL, cm)	7.57	1.17	4.50–12.40	1.06	0.48

**Table 2:** Summary of interval mapping for F_2_ mapping populations derived from two different melon accessions (M202008 and M202009). Chr, chromosome; LOD, logarithm of odds; PVE, phenotypic variation explained; FD, fruit diameter; FL, fruit length.

Year	Trait	Chr	Left Marker	Right Marker	PVE (%)	LOD Value
2023	Fruit diameter (FD)	9	*Chr9_4100141*	*Chr9_5984071*	14.59	5.48
	Fruit length (FL)	9	*Chr9_4100141*	*Chr9_5984071*	16.20	6.16
2024	Fruit diameter (FD)	9	*Chr9_4100141*	*Chr9_6296006*	7.22	6.22
	Fruit length (FL)	9	*Chr9_4100141*	*Chr9_6296006*	6.35	5.44

## Data Availability

The data presented in this study are available upon request from the corresponding author.

## Supplementary Materials

The following supporting information can be downloaded at https://www.mdpi.com/article/10.3390/plants14152254/s1. Table S1: Information on the candidate genes identified in the mapping region; Table S2: Mutations in the coding sequence region of *MELO3C021600.1*; Table S3: Sequence variation in the promoter region of *MELO3C021600.1*; Table S4: Information on the InDel markers used for genetic mapping.
